# Thiamine Deficiency Causes Long-Lasting Neurobehavioral Deficits in Mice

**DOI:** 10.3390/brainsci10080565

**Published:** 2020-08-17

**Authors:** Hui Li, Hong Xu, Wen Wen, Liying Wu, Mei Xu, Jia Luo

**Affiliations:** 1 Department of Pharmacology and Nutritional Sciences, University of Kentucky College of Medicine, Lexington, KY 40536, USA; hui.li@uky.edu (H.L.); hong.xu4@uky.edu (H.X.); wen.wen@uky.edu (W.W.); liying.wu@uky.edu (L.W.); mxu222@uky.edu (M.X.); 2 Lexington VA Health Care System, Research & Development, Lexington, KY 40502, USA

**Keywords:** thiamine deficiency, behavioral test, locomotion, anxiety

## Abstract

Thiamine deficiency (TD) has detrimental effects on brain health and neurobehavioral development, and it is associated with many aging-related neurological disorders. To facilitate TD-related neuropsychological studies, we generated a TD mouse model by feeding a thiamine-deficient diet for 30 days, followed by re-feeding the control diet for either one week or 16 weeks as recovery treatment. We then performed neurobehavioral tests in these two cohorts: cohort of one week post TD treatment (1 wk-PTDT) and 16 weeks post TD treatment (16 wks-PTDT). The TD mice showed no significant difference from control in any tests in the 1 wk-PTDT cohort at the age of 13–14 weeks. The tests for the 16 wks-PTDT cohort at the age of 28–29 weeks, however, demonstrated anxiety and reduced locomotion in TD animals in open field and elevated plus maze. In comparison, rotor rod and water maze revealed no differences between TD and control mice. The current findings of the differential effects of the same TD treatment on locomotion and anxiety at different ages may reflect the progressive and moderate change of TD-induced neurobehavioral effects. The study suggests that, even though the immediate neurobehavioral impact of TD is modest or negligible at a young age, the impact could develop and become severe during the aging process.

## 1. Introduction

Thiamine is an essential vitamin required for normal growth and tissue development. It cannot be synthesized by the human body; therefore, it needs to be supplied in food or as a dietary supplement. The activated form of thiamine, thiamine pyrophosphate or diphosphate (TPP), serves as a cofactor for several key enzymes involved in carbohydrate metabolism. There are many factors that may cause thiamine deficiency (TD): insufficient dietary intake, consumption of anti-thiamine factors, excessive loss of vitamin B1, and chronic alcohol consumption [1,2,3]. If sufficiently prolonged and severe, TD can cause lasting damage to many organ systems, including the cardiac [4], muscular [5], gastrointestinal [6], and nervous system [7]. The brain is highly sensitive to TD, possibly due to its dependence on energy metabolism and biosynthesis of neurotransmitters mediated by thiamine-dependent enzymes [8]. In the brain, TD was shown to cause mild and chronic impairment of oxidative metabolism, neuroinflammation, and neurodegeneration, which are the processes commonly observed in Wernicke–Korsakoff syndrome (WKS) and many aging-related neurodegenerative diseases such as Alzheimer’s disease [9], Parkinson’s disease [10], and progressive supranuclear palsy [11]. Low thiamine levels and depressive symptoms are associated with the aging process [12]. Therefore, animal models for TD are useful for research of WKS and aging-associated degenerative disorders.

There were many studies using rat models of TD induced via feeding of a thiamine-deficient diet or injections of pyrithiamine (PTD), which is a thiamine antagonist, or a combination of both [13,14,15,16,17]. The TD rats were observed with a variety of abnormal behaviors. For example, early studies showed that dietary-induced TD rats exhibited mouse-killing behavior that can be improved by administration of anti-depressants but not by injection of thiamine hydrochloride or a thiamine-supplemented diet, suggesting that the TD-induced muricide response is an irreversible change and can be used as a model for depression [18,19,20]. PTD injection in rats resulted in cognitive and memory deficits on spatial tasks that correlated with neuronal loss in selective brain regions [15]. Impairment of avoidance learning in rats was induced during the course of TD dietary treatment [21,22]. Reduced food intake and loss of body weight was reported in adult rats and developing rats that were fed a TD diet [23]. In addition, TD in rats was observed along with impaired periphery nerves [24,25], changes in heart rate [26,27], altered blood pressure [26], increased startle response to electric shock [28], and a pain threshold (antinociceptive) effect [29].

In contrast to rats, the studies using mouse models for TD are quite limited. The few available studies showed that TD diet-treated mice lost appetite and body weight [30,31,32]. The male mice of the Deutschland, Denken, and Yoken (ddY) stain exhibited increased immobility in the forced swimming test, a depressive behavior observed on the 20th day of treatment of the thiamine-deficient diet [31,33]. The depressive behavior of TD diet-induced ddY mice was associated with monoaminergic neuronal functions in the cortex, amygdala, and brainstem, and it could be rescued by the administration of a Japanese herbal medicine “kami-untan-to”, suggesting that the TD-induced damage is reversible [33]. The dietary-induced TD in mice also displayed an impairment of memory-related behavior during TD diet feeding, and this behavior correlated to the activity of cholinergic neurons in the hippocampus [34]. In addition, TD mice also showed altered circadian rhythmicity [35] and an antinociceptive effect [36].

In this study, we sought to determine whether dietary TD can cause permanent neurobehavioral deficits in mice. We treated adult male C57BL/6 mice with a TD diet for 30 consecutive days, and we then resumed a regular diet for one week (short term) or 16 weeks (long term). After these recovery periods (fed with a regular diet), we performed neurobehavioral tests on the mice, including an open field test, elevated plus maze, rotor rod test, and Morris water maze.

## 2. Materials and Methods

### 2.1. Models of Thiamine Deficiency

The male C57BL/6 mice of seven weeks old were purchased from Jackson Laboratories (Bar Harbor, ME, USA). Animals were acclimated to housing facilities for at least one week prior to the beginning of the experiment, at which time they were approximately eight weeks old. Mice were maintained on a 14-h/10-h light/dark cycle (lights on at 7:00 a.m., off at 9:00 p.m.), group-housed (typically four to five in each cage), and always given free access to water. All procedures described were approved by the Institutional Animal Care and Use Committee (IACUC) at the University of Kentucky.

Two cohorts of animals were used to address the effect of thiamine deficiency (TD): cohort of one week post TD treatment (1 wk-PTDT cohort, *n* = 10 for both control and TD groups) and cohort of 16 weeks post TD treatment (16 wks-PTDT cohort, *n* = 10 for control group and *n* = 9 for TD group). There were two experimental groups, a control and TD group, and the number of animals in each group in each cohort was indicated. Animals were randomly assigned to either the control or the TD group. Mice in the control group were fed a control diet (MP Biomedicals LLC, Irvine, CA, USA, catalog no. 0296042010) throughout the treatment, whereas animals in the TD group were fed a TD diet (MP Biomedicals LLC, Irvine, CA, USA, catalog no. 0296016510) for 30 consecutive days until they displayed significant body weight loss; then, they were resumed on the control diet. The nutrient analysis showed that thiamine hydrochloride is missing in the TD diet in comparison with the control diet. Animals were subjected to behavioral tests either one week after the treatment (1 wk-PTDT cohort) or 16 weeks after the treatment (16 wks-PTDT cohort). All behavioral tests were performed in the Rodent Behavior Core (RBC) at the University of Kentucky following standard procedure. The study was approved by the University of Kentucky IACUC (2008-0401) on 10 November 2019.

### 2.2. Open Field Test

The open field (OF) test is one of the most common behavioral assays to measure locomotor activity and anxiety-like behaviors in rodents [37]. The test was conducted repeatedly for each animal for 15 min each day for two consecutive days utilizing a cubic box measured 50 cm (length) × 50 cm (width) × 38 cm (height) in a multi-unit open field arena (San Diego Instruments, San Diego, CA, USA). Before the test, animals were brought to the testing room to acclimate for 10 min as a habituation period. Each animal was then placed on the bottom surface, and its activity was recorded using EthoVision XT 8.0 video-tracking software (Noldus Information Technology, Leesburg, VA, USA). The time spent in various regions of the open field (s) and the total distance traveled (cm) were recorded. The center zone was defined as the central area measured 25 cm (length) × 25 cm (width). Distance traveled was a measure of locomotor activity, while the time spent in the center was a measure of anxiety.

### 2.3. Rotor Rod Test

The Rotor rod test was applied as described previously [38]. The rotor rod system allows using an animal’s natural fear of falling instinct as a motivator to study motor coordination. Each animal is placed on a rotating rod with a diameter 1.25″ × 4.5″ wide and 18” above the ground in the rotor rod apparatus with dimensions of 33” (H) × 36” (W) × 24” (D) (San Diego Instruments, San Diego, CA, USA). The rod was rotated from a speed of 4 rpm to 40 rpm over the course of 5 min. The latency to fall, which is the cumulative time (s) that each animal remained on the rotating rod, was recorded as an indicator of its motor coordination.

### 2.4. Elevated Plus Maze

The elevated plus maze (EPM) is one of the most used tests for anxiety-related behavior in rodents [39], and it is believed to be the most sensitive assay for anxiety [40]. The EPM apparatus consisted of a cross-shaped maze which was 50 cm above the ground with two opposite open arms and two opposite closed arms surrounded by 25-cm-high dark walls, and each arm measured 30 cm (length) × 6 cm (width). Each animal was tested for 5 min to freely explore the maze. The movements of each animal were video-recorded and computer-analyzed with EthoVision XT 8.0 video-tracking software (Noldus Information Technology, Leesburg, VA, USA). The percentage of time spent in each arm and the entries into each arm were analyzed.

### 2.5. Morris Water Maze

The Morris water maze (MWM) is a test of spatial learning and memory for rodents [41,42]. Animals undergo training and testing sessions include spatial learning, probe trials, reversal learning, and visible cue. The MWM apparatus was a round tank with a diameter of 108 cm filled with white-painted water. A circular platform was placed about 1 cm below the water surface in one of the quadrants. Four distinct cues were attached to the walls around the tank. Each animal was placed at random starting locations in the quadrant distal to the platform and allowed to swim to find the submerged platform. If the platform was not located within one minute, animals were gently guided to it, and they remained on the platform for 10 s to re-orient to the surrounding cues. For spatial learning, each mouse was given four daily trials for five consecutive days. To examine spatial reference memory, the platform was removed, and a single probe trial was conducted 24 h after the last training session. Each mouse was placed in the opposite quadrant and allowed to swim freely for 1 min. Four hours after the probe trial, the platform was placed in the opposite quadrant, and each mouse was tested in four trials for reversal learning. Following the reversal learning, a single visible cue test was conducted with the platform marked by a visible rod above the water surface. The visible cue test was applied for potential visual deficits as a result of thiamine deficiency. All tracks from all trials were recorded and analyzed using EthoVision XT 8.0 video-tracking software (Noldus Information Technology, Leesburg, VA, USA).

### 2.6. Statistical Analysis

The data were expressed as mean ± standard error of the mean (SEM). The statistical analyses were performed using SPSS software 19 (IBM, Armonk, NY, YSA) and Graphpad Prism 6 (San Diego, CA, USA). One-way analysis of variance (ANOVA) was performed with treatment and day as grouping factors and repeated measures when warranted. The Greenhouse–Geisser correction was used to correct the F statistic and assess significance when necessary. A *p*-value of 0.05 was considered significant.

## 3. Results

### 3.1. Body Weight

**Figure 1:**
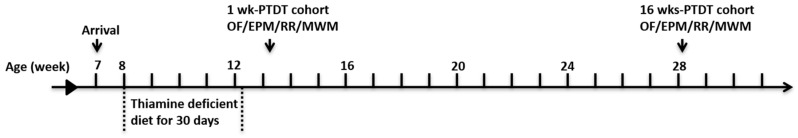
Schematic representation of the timeline of thiamine deficiency (TD) treatment and behavioral tests for the two cohorts of male C57BL6 mice. The behavioral tests were performed in the order of open field (OF), elevated plus maze (EPM), rotor rod (RR), and Morris water maze (MWM) either one week post TD treatment (1 wk-PTDT cohort) or 16 weeks post TD treatment (16 wks-PTDT cohort). For the 1 wk-PTDT cohort, *n* = 10 for both control and TD groups. For the 16 wks-PTDT cohort, *n* = 10 for control group, and *n* = 9 for TD group.

**Figure 2:**
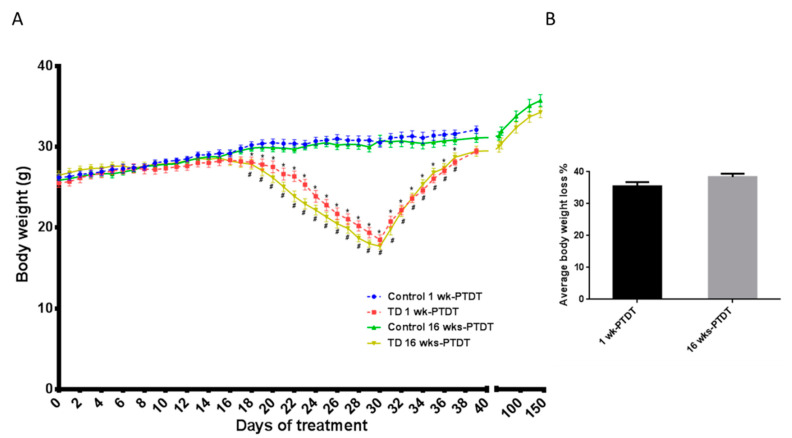
The effects of thiamine deficiency (TD) on body weight. The body weight profiles of animals in each group in the two cohorts were recorded (**A**), and the maximal body weight loss during TD treatment was calculated as the ratio between the body weight loss on the 30th day of TD treatment and the maximal body weight the animal achieved, which occurred mostly at around the 14th day of TD treatment (**B**). * *p*  <  0.05 denotes a statistically significant difference from the control group in the 1 wk-PTDT cohort; # *p*  <  0.05 denotes a statistically significant difference from the control group in the 16 wks-PTDT cohort. For the 1 wk-PTDT cohort, *n* = 10 for both control and TD groups. For the 16 wks-PTDT cohort, *n* = 10 for the control group, and *n* = 9 for the TD group.

To determine the effects of TD on behavioral tests, two cohorts of animals were used in this study: cohort of one week post thiamine deficiency treatment (1 wk-PTDT cohort) and cohort of 16 weeks post thiamine deficiency treatment (16 wks-PTDT cohort). The timelines of TD treatment and behavioral tests for both cohorts are presented in Figure 1. In each cohort, the body weight of animals was recorded as presented in Figure 2A. The average body weights of each group in both cohorts were similar at the beginning of the experiments, and there was no significant difference (F_(3, 35)_ = 0.5779, *p* = 0.6334): control of 1 wk-PTDT = 26.3 ± 0.4 g (mean + SEM); TD of 1 wk-PTDT = 25.7 ± 0.5 g; control of 16 wks-PTDT = 25.9 ± 0.4 g; TD of 16 wks-PTDT = 26.5 ± 0.5 g. In both cohorts, the control and TD mice gained body weight during the first 10 days of the TD diet. After two weeks, the TD animals started losing body weight rapidly until the 30th day, when the TD diet was replaced with a regular diet. On the 30th day of treatment in the 1 wk-PTDT cohort, the average body weight of the TD animals was reduced to 18.5 ± 0.6 g, which was significantly lower than in the control animals (30.5 ± 0.5 g) (*t*(18) = 14.62, *p* < 0.001). Similarly, there was a significant difference (*t*(17) = 16.85, *p* < 0.001) between the average body weight of the TD mice (17.7 ± 0.4 g) and the control mice (30.8 ± 0.7 g) in the 16 wks-PTDT cohort on day 30. As expected, the repeated-measures ANOVA analysis of body weight during the TD treatment for the 1 wk-PTDT cohort demonstrated significant main effects of treatment (F(1,18) = 23.813, *p* < 0.001) and day (F(2.537,45.664) = 378.931, *p* < 0.001). The treatment by day interaction was also significant (F (2.537,45.664) = 114.616, *p* < 0.001_. Further statistical analysis demonstrated a significant difference between the average body weight of control and TD groups starting from the 18th day for the 1 wk-PTDT cohort (F_1-wk-PTDT, day 17_ (1, 18) = 3.978, *p* = 0.061; F_1-wk-PTDT, day 18_ (1, 18) = 6.972, *p* = 0.017; F_1-wk-PTDT, day 19_ (1, 18) = 10.422, *p* = 0.005). Meanwhile, the ANOVA analysis for the 16 wks-PTDT cohort showed that there was a significant main effect of treatment (F(1, 17) = 30.080, *p* < 0.001) and day (F(2.402, 40.827) = 57.202, *p* < 0.001), as well as treatment by day interaction (F(2.402, 40.827) = 57.202, *p* < 0.001). Similar to the 1 wk-PTDT cohort, further analysis indicated that the average body weight significantly differed between the control and TD groups starting from the 18th day for the 16 wks-PTDT cohort (F_16-wks-PTDT, day 17_(1, 17) = 3.302, *p* = 0.087; F_16-wks-PTDT, day 18_ (1, 17) = 9.570, *p* = 0.007; F_16-wks-PTDT, 19th day_ (1, 17) = 13.240, *p* = 0.002). The body weight loss of the TD animals during the period of 30-day TD treatment ranged from 28.4% to 40.7% for the 1 wk-PTDT cohort and 31.8% to 42.5% for the 16 wks-PTDT cohort. Further calculation indicated that the average body weight loss was 35.5% ± 1.3% (*n* = 10) for the 1 wk-PTDT cohort, which was not significantly different from that for the 16 wks-PTDT cohort (38.4% ± 1.1%, *n* = 9) (*t* (17) = 1.717, *p* = 0.1042) (Figure 2B).

The loss of body weight was gradually recovered after resuming the regular diet. One week after being fed with the regular diet, the average body weight of TD animals in the 1 wk-PTDT cohort was 28.1 ± 0.3 g, which was still significantly lower than that of the control animals 31.6 ± 0.5 g (*t*(18) = 5.89, *p* < 0.001). Sixteen weeks after being fed a regular diet, the average body weight of the TD group of the 16 wks-PTDT cohort (34.3 ± 0.7 g) was comparable to that of the control group (35.7 ± 0.7 g) [*t*(17) = 1.493, *p* = 0.1539].

### 3.2. Open Field Test

**Figure 3:**
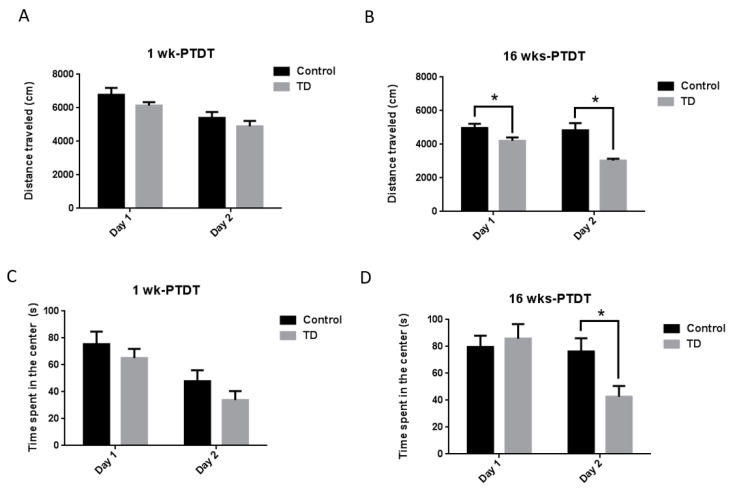
The results of the open field (OF) test of the two cohorts after TD treatment. The total distance travelled for two consecutive days was calculated for the 1 wk-PTDT cohort (**A**) and the 16 wks-PTDT cohort (**B**). The time spent in the center was also recorded for the 1 wk-PTDT cohort (**C**) and the 16 wks-PTDT cohort (**D**). For the 1 wk-PTDT cohort, *n* = 10 for both control and TD groups. For the 16 wks-PTDT cohort, *n* = 10 for the control group, and *n* = 9 for the TD group. * *p*  <  0.05 denotes a statistically significant difference from the control group.

We firstly performed the open field test, which is one of the most commonly used tests to measure locomotor activity and anxiety-like behaviors in rodents [37,43]. The locomotor activity was measured using the total distance traveled (cm) and the anxiety-like level was measured using the time spent in the center (s). For the 1 wk-PTDT cohort, the total distance on day 1 was 6784.7 ± 408.9 cm for the control mice and 6141.8 ± 200.8 cm for TD mice, while it was 5403.9 ± 347.1 cm for the control mice and 4904.6 ± 316.7 cm for the TD mice on day 2 (Figure 3A). The statistical analysis demonstrated a significant main effect of day (F(1, 18) = 70.438, *p* < 0.001) and insignificant main effect of treatment (F(1, 18) = 1.719, *p* = 0.206). However, the treatment by day interaction was not significant (F(1, 18) = 0.212, *p* = 0.651). The time spent in the center was 75.4 ± 9.4 s for the control animals and 65.1 ± 6.9 s for TD animals on day 1, while it was 47.9 ± 8.1 s for the control animals and 33.9 ± 6.6 s for TD animals on day 2 (Figure 3C). The statistical analysis showed there was a significant main effect of day (F(1, 18) = 52.013, *p* < 0.001) but not treatment (F(1, 18) = 1.396, *p* = 0.253). The treatment by day interaction was not significant (F(1, 18) = 0.215, *p* = 0.649). For the 16 wks-PTDT cohort, the total distance on day 1 was 4972.3 ± 250.6 cm for the control and 4214.9 ± 196.7 cm for the TD group, while it was 4834.4 ± 425.8 cm for the control and 3027.8 ± 116.6 cm for the TD group on day 2 (Figure 3B). The statistical analysis demonstrated a significant main effect of day (F(1, 17) = 19.801, *p* < 0.001) and treatment (F(1, 17) = 11.945, *p* = 0.003), as well as the treatment by day interaction [F(1, 17) = 12.42, *p* = 0.003]. Further analysis showed that there was a significant difference between the total distance on both day 1 (F(1, 17) = 5.474, *p* = 0.032) and day 2 (F(1, 17) = 15.199, *p* = 0.001). The time spent in the center was 79.6 ± 8.5 s for the control and 85.9 ± 10.7 s for the TD group on day 1, while it was 76.3 ± 9.8 s for the control and 42.7 ± 7.9 s for the TD group on day 2 (Figure 3D). The statistical analysis indicated that the main effect of day (F(1, 17) = 6.830, *p* = 0.018) was significant, whereas the main effect of treatment (F(1, 17) = 1.972, *p* = 0.178) was not significant. The treatment by day interaction was significant (F(1, 17) = 5.024, *p* = 0.039). Further analysis indicated that there was a significant difference between the time spent in the center on day 2 (F(1, 17) = 6.883, *p* = 0.018) but not on day 1 (F(1, 17) = 0.219, *p* = 0.646).

### 3.3. Elevated Plus Maze Test

**Figure 4:**
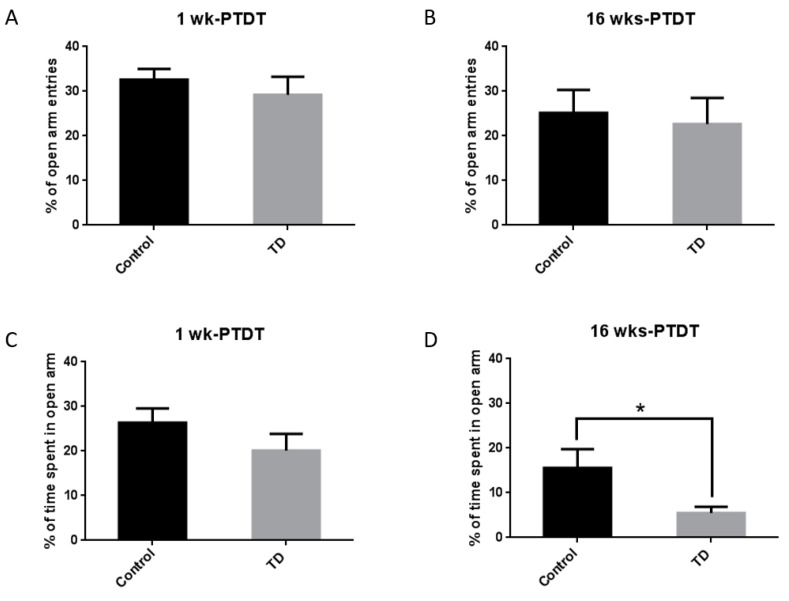
The results of the elevated plus maze (EPM) test of the two cohorts after TD treatment. The percentage of open-arm entries was calculated for the 1 wk-PTDT cohort (**A**) and the 16 wks-PTDT cohort (**B**). The percentage of time spent in open arms was determined for the 1 wk-PTDT cohort (**C**) and the 16 wks-PTDT cohort (**D**). For the 1 wk-PTDT cohort, *n* = 10 for both control and TD groups. For the 16 wks-PTDT cohort, *n* = 10 for the control group, and *n* = 9 for the TD group. * *p*  <  0.05 denotes a statistically significant difference from the control group.

The elevated plus maze (EPM) is a widely used test for anxiety-related behaviors in rodents [39]. The percentage of entries and time spent in the open arms were measured as indicators for anxiety level. For the 1 wk-PTDT cohort, the percentage of open-arm entries was 32.6% ± 2.4% for the control and 29.2% ± 4.0% for the TD group, and there was no significant difference (*t*(18) = 0.7269, *p* = 0.4767) (Figure 4A). The percentage of time spent in the open arm was 26.4% ± 3.2% for the control and 20.1% ± 3.8% for the TD group, and there was no significant difference (*t*(18) = 1.263, *p* = 0.2227) (Figure 4C). For the 16 wks-PTDT cohort, there was no significant difference in the percentage of open-arm entries in the control group (25.2% ± 5.1%) and TD group (22.7% ± 5.8%) (*t*(17) = 0.3224, *p* = 0.7511) (Figure 4B). However, there was a significant difference in the percentage of time spent in the open arm (*t*(17) = 2.198, *p* = 0.0421) between the control group (15.6% ± 4.2%) and TD group (5.5% ± 1.4%) (Figure 4D).

### 3.4. Rotor Rod Test

**Figure 5:**
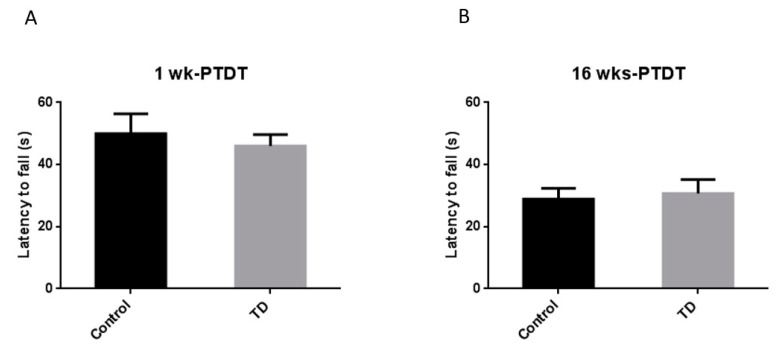
The results of the rotor rod (RR) test of the two cohorts after TD treatment. The latency to fall was recorded for the 1 wk-PTDT cohort (**A**) and the 16 wks-PTDT cohort (**B**). For the 1 wk-PTDT cohort, *n* = 10 for both control and TD groups. For the 16 wks-PTDT cohort, *n* = 10 for the control group, and *n* = 9 for the TD group.

The rotor rod test is used to assess the motor coordination of rodents [44]. The latency to fall (s) was recorded for each animal and compared. For the 1 wk-PTDT cohort, the latency to fall was 50.0 ± 6.4 s for the control group and 46.0 ± 3.7 s for the TD group. There was no significant difference (*t*(18) = 0.5368, *p* = 0.598) (Figure 5A). For the 16 wks-PTDT cohort, there was no significant difference in the latency to fall between the control group (28.9 ± 3.5 s) and TD group (30.7 ± 4.5 s) (*t*(17) = 0.3343, *p* = 0.7422) (Figure 5B). These results indicated the TD had no effect on motor coordination in either cohort.

### 3.5. Morris Water Maze

**Figure 6:**
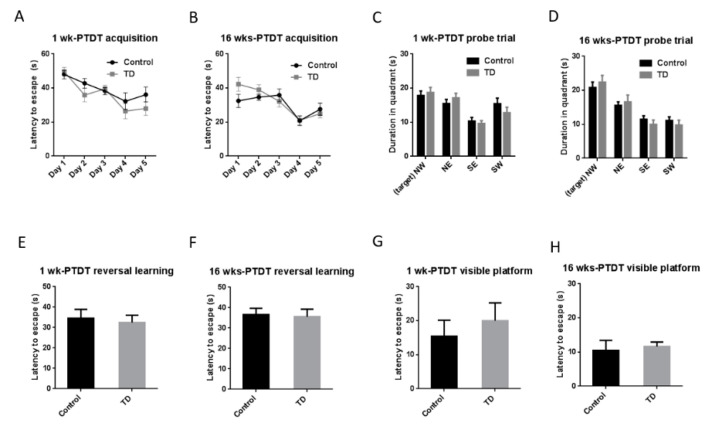
The results of the Morris water maze (MWM) test of the two cohorts after TD treatment. The acquisition time in the 1 wk-PTDT cohort (**A**) and the 16 wks-PTDT cohort (**B**); the duration in each quadrant in the probe trial in the 1 wk-PTDT cohort (**C**) and the 16 wks-PTDT cohort (**D**) (NW: northwest quadrant where the platform was placed; NE: northeast; SE: southeast; SW: southwest); the latency to escape during reversal learning in the 1 wk-PTDT cohort (**E**) and the 16 wks-PTDT cohort (**F**); the latency to escape during the visible platform test in the 1 wk-PTDT cohort (**G**) and the 16 wks-PTDT cohort (**H**). For the 1 wk-PTDT cohort, *n* = 10 for both control and TD groups. For the 16 wks-PTDT cohort, *n* = 10 for the control group, and *n* = 9 for the TD group. The Graphpad Prism *t*-test and one-way repeated-measures ANOVA were performed when necessary.

The Morris water maze is a test of spatial learning and memory for rodents [41]. The spatial acquisition for the 1 wk-PTDT cohort (Figure 6A) showed that there was a significant main effect of day (F(4, 72) = 9.164, *p* < 0.001) but not treatment (F(1, 18) = 2.504, *p* = 0.131). Similarly, the acquisition for the 16 wks-PTDT cohort demonstrated that there was a significant main effect of day (F(4, 68) = 12.191, *p* < 0.001) but not treatment (F(1, 17) = 0.378, *p* = 0.547) (Figure 6B). For the probe trial, the statistical analysis showed there was no significant difference between the control group (17.84 ± 1.335 s) and TD group (18.74 ± 1.491 s) (*t*(18) = 0.4517 *p* = 0.6569) in the 1 wk-PTDT cohort (Figure 6C). In the 16 wks-PTDT cohort, there was no significant difference (*t*(17) = 0.6133, *p* = 0.5478) between the control group (20.84 ± 1.599 s) and TD group (22.39 ± 1.989 s) (Figure 6D). The results of reversal learning are shown in Figure 6E and 6F. There was no significance (*t*(18) = 0.3678, *p* = 0.7173) between the control (34.55 ± 4.345 s) and the TD group (32.48 ± 3.555 s) in the 1 wk-PTDT cohort (Figure 6E). In the 16 wks-PTDT cohort, the reversal learning of the control (36.65 ± 3.090 s) was not significantly different from the TD group 35.58 ± 3.684 s (*t*(17) = 0.2238, *p* = 0.8256) (Figure 6F). The additional visible platform test indicated there was no significant difference (*t*(18) = 0.6451 *p* = 0.5270) between the control group (15.44 ± 4.731 s) and TD group (20 ± 5.253 s) in the 1 wk-PTDT cohort (Figure 6G). In the 16 wks-PTDT cohort, the visibility of the control group (10.51 ± 2.931 s) was not significantly different (*t*(17) = 0.3347, *p* = 0.7420) from the TD group (11.63 ± 1.330 s) (Figure 6H) either. Collectively, these observations indicated that TD did not impair memory performance and learning.

## 4. Discussion

Thiamine is an essential vitamin required for the metabolism of glucose, lipids, and amino acids. In humans, TD can result from inadequate intake of thiamine from food, lower absorption, alcohol abuse, or higher excretion rates due to certain conditions. It is a medical condition manifested in the early stages by confusion, loss of appetite, and ataxia. It can persist in a chronic state characterized by memory disorders such as amnesia and short-term memory loss. In this study, we used a thiamine-deficient diet to feed adult male C57BL6 mice for 30 days continuously to mimic dietary-induced TD, and we examined the acute and chronic effects of TD in a set of behavioral tests. The TD animals in two cohorts, 1 wk-PTDT and 16 wks-PTDT cohort, displayed a similar body weight loss profile during the TD dietary feeding period, which indicated that all mice responded equally to the treatment of TD over the 30 days of induction, allowing a comparison of their behaviors. The TD animals in the 1 wk-PTDT cohort at the age of 13–14 weeks (equivalent to a human at 20 years old) showed no difference in the behavioral tests from the control animals, suggesting no acute effect of TD in young animals. In contrast, the TD animals in the 16 wks-PTDT cohort at the age of 28–29 weeks (equivalent to a human at 35–40 years old) displayed reduced locomotor activity and anxiety.

Locomotor activity is a critical physiological function in humans. Patients with TD neuropathy were observed with limited locomotion [45,46]. In a rat model of stress, thiamine supplementation was shown to increase the locomotor activity of stressed rats by regulating the brain-derived neurotrophic factor and acetylcholine in the hippocampus [47]. Adult male Swiss mice, when treated with thiamine antagonist pyrithiamine in conjunction with a TD diet, showed a reduction in locomotor activity during the course of dietary treatment [48]. Another thiamine antagonist, amprolium, also decreased the locomotor activity of mice when either treated alone with a high dose and extended period [49] or combined with a TD diet [50]. In our model, we only used a TD diet without adding a thiamine antagonist. This approach gradually reduced thiamine concentration in the body, which produced a milder impact and better model of human TD condition. The locomotor activity, which was measured as the total distance travelled in the open field, however, was different for the TD animals in both cohorts. The TD animals in the 1 wk-PTDT cohort at the age of 13–14 weeks travelled 9.5% less on day 1 and 9.2% less on day 2 than the control animals. The result, however, was not considered statistically significant (*p* = 0.206). In the 16 wks-PTDT cohort, when the animals were 28–29 weeks old, the locomotor activity of TD animals was reduced by 15.2% on day 1 and 37.4% on day 2 compared with the control, and the result was considered statistically significant on both days (*p_day 1_* = 0.032; *p_day 2_* = 0.001). Taken together, the results suggested that the young mice were more resistant to the adverse effect of TD on locomotor activity. However, the effect of TD may manifest when the mice get older. This may be of interest because aging-related decline in human locomotor performance is widely documented [51,52,53,54,55], as well as TD in elderly patients [56,57,58]. The findings of this study may implicate that the occurrence of TD at a young age, if not treated in a timely manner, may lead to in locomotion deficit in elderly patients.

Thiamine is used to treat patients with anxiety, and it was shown to improve many of the symptoms, suggesting an anxiolytic effect of thiamine [59,60]. Thiamine supplementation ameliorated the anxiety-like behavior of stressed-induced rats [47] and stress-induced mice [61]. TD causes symptoms including fatigue, anorexia, and nausea, which are also observed in anxiety disorders. A TD rat model induced by a 37-day TD diet exhibited anxiety-like behavior [62]. In this study, anxiety level was measured as the time animals spent in the center in the open field and the percentage of open arm entries/time in the elevated plus maze. In the 1 wk-PTDT cohort, when the animals were at the age of 13–14 weeks, the TD group spent less time (reduction by 13.7% and 29.2% on day 1 and 2, respectively) in the center of the open field (F(1,18) = 1.396, *p* = 0.253) and less time in the open arm (reduction by 23.9%) in the elevated plus maze (*t*(18) = 1.263, *p* = 0.2227), but none of the results were statistical significant. In the 16 wks-PTDT cohort, when the animals were at the age of 28–29 weeks, the statistical analysis indicated that the TD group spent significantly less time (reduction by 44.0%) in the center of the open field on day 2 (*p* = 0.018, Figure 3D) and significantly less time in the open arm in the elevated plus maze (reduction by 64.6%, *p* = 0.0421, Figure 4D). Similar to locomotion performance, young animals were resistant to TD-induced anxiety and asymptomatic. However, when they became older, the impact of TD on anxiety was displayed, suggesting that TD has a long-lasting effect on neurobehavioral outcomes.

The cerebellum-mediated motor coordination appears to be insensitive to TD-induced damage from previous publications. Nakagawasai et al. generated TD mice by feeding male ddY mice with a TD diet for 20 consecutive days, and they measured the motor coordination on the 20th day [31]. They showed that there was no difference between the TD animals and control animals. This was probably due to the mild nature of the TD dietary treatment. However, a much more severe approach using a TD diet combined with daily injections of thiamine antagonist pyrithiamine for 10 days caused shorter latency to fall in the mice during the rotor rod test, and this motor coordination deficit was rescued by a single injection of thiamine, showing that TD-induced motor incoordination is reversible [38]. In our study, the motor coordination, measured as the latency to fall in the rotor rod, was not affected in TD animals in either cohort. The insensitivity of motor coordination to TD-induced lesions may reflect the remarkable capacity of the cerebellum to compensate and restore its functions [63,64,65] and the mild nature of our TD model. Motor coordination becomes more vulnerable during the aging process. Therefore, in future study, it would be desirable to evaluate motor coordination in aged animals (e.g., 72–96 weeks old, equivalent to a human of 60–70 years old).

There were reports on the association between TD and memory impairments in animals; the studies were mainly performed in rat models. TD rats showed enduring impairment in passive avoidance learning [15,22,62], the water maze task [66,67], and the T-maze test [15,68,69]. In mice, the impairment of learning and memory was observed during the period animals were fed a TD diet, suggesting an acute effect of TD [34,70]. More severe approaches, such as a TD diet in combination with either pyrithiamine [38] or chronic alcohol drinking [71], could produce long-term memory impairments in mice. In our study, the TD animals in both cohorts did not show any behavioral deficits in the Morris water maze after a recovery period of one week or 16 weeks, indicating that the lesions induced by TD diet alone may not be severe enough to cause long-lasting change in learning and memory. Alternatively, the deficits may manifest when animals are aged. Therefore, it would be interesting to evaluate learning and memory in aged animals (e.g., 70–90 weeks old) in future study.

One of the limitations of this study was the failure to examine the TD-induced immediate effects on neurobehaviors during the feeding period. As suggested by previous publications, some of the TD-induced deficits, such as loss of motor coordination [38] and impaired learning and memory [34,70], were observed during or immediately after the feeding period. It may also be of interest to determine the immediate effects of TD on locomotion and anxiety.

In summary, the TD mouse model established in this study may be useful to assess TD-induced neuropathology in preclinical studies. The effects on locomotion and anxiety became most evident after 16 weeks but not one week of treatment, reflecting a progressive and moderate manner of TD-induced damage, which may recapitulate some characteristic features observed in human TD. The finding suggests that, although young animals are more resistant to the impact of TD and are asymptomatic given a period of recovery, the long-lasting effect of TD may manifest when they became older. The long-term effects of thiamine deficiency, alone or combined with alcohol exposure, were documented in humans [72,73] or rats [71,74]. In addition, this model allows TD-induced damage to progressively develop, which will be valuable in the study of molecular mechanisms underlying the interaction between aging and TD-induced neuropathology.
